# Impact of using time-averaged exposure metrics on binary endpoints in exposure-response analyses

**DOI:** 10.3389/fphar.2024.1487062

**Published:** 2025-01-16

**Authors:** Yu-Wei Lin, Anna Largajolli, A. Yin Edwards, S. Y. Amy Cheung, Kashyap Patel, Stefanie Hennig

**Affiliations:** ^1^ Certara Inc., Melbourne, VIC, Australia; ^2^ Monash Biomedicine Discovery Institute, Infection Program and Department of Microbiology, Monash University, Clayton, VIC, Australia; ^3^ Centre for Medicine Use and Safety, Faculty of Pharmacy and Pharmaceutical Sciences, Monash University, Parkville, VIC, Australia; ^4^ Malaya Translational and Clinical Pharmacometrics Group, Faculty of Pharmacy, Universiti Malaya, Kuala Lumpur, Malaysia; ^5^ Department of Clinical Pharmacy and Pharmacy Practice, Faculty of Pharmacy, Universiti Malaya, Kuala Lumpur, Malaysia; ^6^ Certara Inc., Princeton, NJ, United States; ^7^ Radnor Corporate Center, Radnor, PA, United States; ^8^ School of Clinical Sciences, Faculty of Health, Queensland University of Technology, Brisbane, QLD, Australia

**Keywords:** drug development, exposure-response analysis, exposure metrics, pharmacometrics, logistic regression

## Abstract

Exposure-response (ER) analyses are routinely performed as part of model-informed drug development to evaluate the risk-to-benefit ratio for dose selection, justification, and confirmation. For logistic regression analyses with binary endpoints, several exposure metrics are investigated, based on pharmacological plausibility, including time-averaged concentration to event (C_avTE_). C_avTE_ is informative because it accounts for dose interruptions, modifications, and reductions and is therefore often compared against ER relationships identified using steady-state exposures. However, its derivation requires consideration in a logistic regression framework for time-invariant ER analysis because it has the potential to introduce bias. This study evaluated different approaches to derive C_avTE_ for subjects whom did not have an event by the end of treatment (EoT) and assessed their impact on the ER relationship. Here we used a modified model based on a real data example for simulating exposures and events (safety) in different virtual population sizes (n = 50, 100, or 200) and drug effect magnitudes (0.5, 0.75, or 1). Events were generated using a proportional odds model with Markov components. For subjects whom did not experience an event, C_avTE_ was derived at EoT, EoT+7 days, +14 days, +21 days, +28 days. The derivation of C_avTE_ at different time points demonstrated significant impact on trends detected in logistic ER relationships that could bias subsequent event projection, dose selection and Go/No-Go decisions. C_avTE_ in censored subjects must therefore be carefully derived to avoid potentially making false positive or negative conclusions. Overall, C_avTE_ can be a useful exposure metrics in an ER analysis, when considered along with physiological or biological plausibility, the drug’s pharmacokinetic, and mechanism of action. Biological plausibility and different analysis factors (e.g., the time of the events with respect to observational period, the level of dose reduction/interruption) should be considered in the choice of the exposure metric. It is recognized that although time-invariant logistic regression is relatively fast and efficient, it overlooks recurring events and does not take into account the exposure and response time course with the potential drawback of ignoring important elements of the analysis like onset or duration of the effect. Care should be taken when ER relationships with other exposure metrics do not identify any statistically significant trends.

## 1 Introduction

Exposure-response (ER) analyses are routinely performed in drug development to evaluate the risk-to-benefit ratio, primarily to inform decisions around dose selection, justification, and confirmation (Ruiz-Garcia et al., 2023). ER models are commonly employed from phase 1–3 onwards, and support the learning-confirming paradigm in drug development (Overgaard et al., 2015; Yee et al., 2019; Wang et al., 2022; Hu et al., 2018). For event analyses, whether logistic regression with binary endpoints or survival analysis with time-to-event (TTE) endpoints (e.g., progression-free survival), the choice and derivation of exposure metric may influence key decisions during ER model development. Examples of binary endpoints include the objective response rate for efficacy or treatment-emergent adverse events (AEs) for safety. Typically, several exposure metrics are selected when investigating ER relationships. The most common exposures that are suggested in regulatory guidance documents (Overgaard et al., 2015; Food and Drug Administration, 2003) include the maximum concentration, minimum concentration, and area under the concentration-time curve (AUC) after the first dose or cycle 1 (e.g., for oncology drugs), or at SS. Additional exposures used in assessing ER include the average concentration at steady-state (SS, C_av,ss_) (Yin et al., 2021a) and time-averaged concentration to event (C_avTE_) (Yin et al., 2021b). C_av,ss_ is a metric that describes the exposure over a given dosing interval, and as such can be linked to responses after chronic treatment. This, together with the AUC at SS (AUC_ss_) has been a preferred exposure metric in ER analyses. Recently, C_avTE_ is also more frequently requested by regulators for comparison against SS exposures, since it accounts for dose modifications, interruptions, or drug withdrawal. Although C_avTE_ can more accurately reflect the actual exposure related to dose changes, careful consideration is required when applying it to a logistic regression framework of time-invariant ER analysis, which generally considers the first occurrence of the event grade of interest. Overall, the choice of the exposure metric should be based on physiological or biological plausible reasons and adapted towards the endpoint of interest (Ruiz-Garcia et al., 2023).

In standard ER analyses, exposure metrics are derived using individual empirical Bayes estimates from a developed population pharmacokinetic (PopPK) model. For each subject, the PopPK model is then applied to simulate and predict concentrations using an intensive sampling design. This process allows for accurate derivation of individual exposure metrics that are relevant to ER analyses. Commonly derived exposure metrics and their definitions, as used in this paper, are as follows:

• minimum concentration at the end of a dosing interval at SS,
• maximum concentration achieved during a dosing interval at SS,
• AUC_ss_–cumulative concentration within a dosing interval at SS, alternatively AUC_ss_ is defined by Dose × Bioavailability/Clearance (CL),
• C_av,ss_–AUC_ss_ divided by the dosing interval,
• C_avTE_–cumulative AUC since start of treatment up to an event divided by time to an event since start of treatment.

C_avTE_ is generally computed as time-averaged exposure using the actual observed dosing history (e.g., to account for missing dose), where time is the time of the measured event. Consequently, C_avTE_ accurately reflects the individual’s average concentration at the time of an event based on actual rather than nominal dosing. The derivation of C_avTE_ is challenging in those subjects that either do not experience an event or are lost at follow-up. For these subjects, the event time is absent and is therefore defined as censored. C_avTE_ in censored subjects is commonly derived using either time at the end of treatment (EoT), EoT + follow-up time per protocol, or the data cut-off date. In all cases, the time of the ‘non-event’ is not available and therefore requires some method of imputation to derive C_avTE_. Based on the definition of C_avTE_ above, it is noted that imputed time can influence ER relationships that include subjects without events (Supplementary Figure S1). We initially highlighted the potential for bias using a case study in 2022 (Patel et al., 2022) where time imputation was varied when deriving C_avTE_ in censored subjects. Others have described different types of bias, (such as election bias and immortal time effects) that need consideration in ER modelling (Khandelwal et al., 2022), and have shown a similar impact when looking at censored subjects (Wiens et al., 2023).

An alternative approach to handling event type data is TTE survival analysis, which accounts for censoring. While both methods investigate the association between drug exposure and events, logistic regression focuses on the dose-exposure-response relationships without taking the event time into consideration. Logistic regression ER analyses are currently more commonly used to support dose finding in drug development programs, with the aim of identifying the optimal therapeutic dose that minimizes risk (safety) and yet maximizes efficacy. Whilst logistic regression ER models are simpler to implement than TTE models, several assumptions or methodological limitations must be considered (Khandelwal et al., 2022).

This case report illustrates the impact of applying different derivations of C_avTE_ in subjects without events on modelled ER relationships within a logistic regression framework. We used a modified real case example to demonstrate the potential introduction of bias, depending on the methods used for time imputation.

## 2 Methods

### 2.1 Motivation example

**FIGURE 1:**
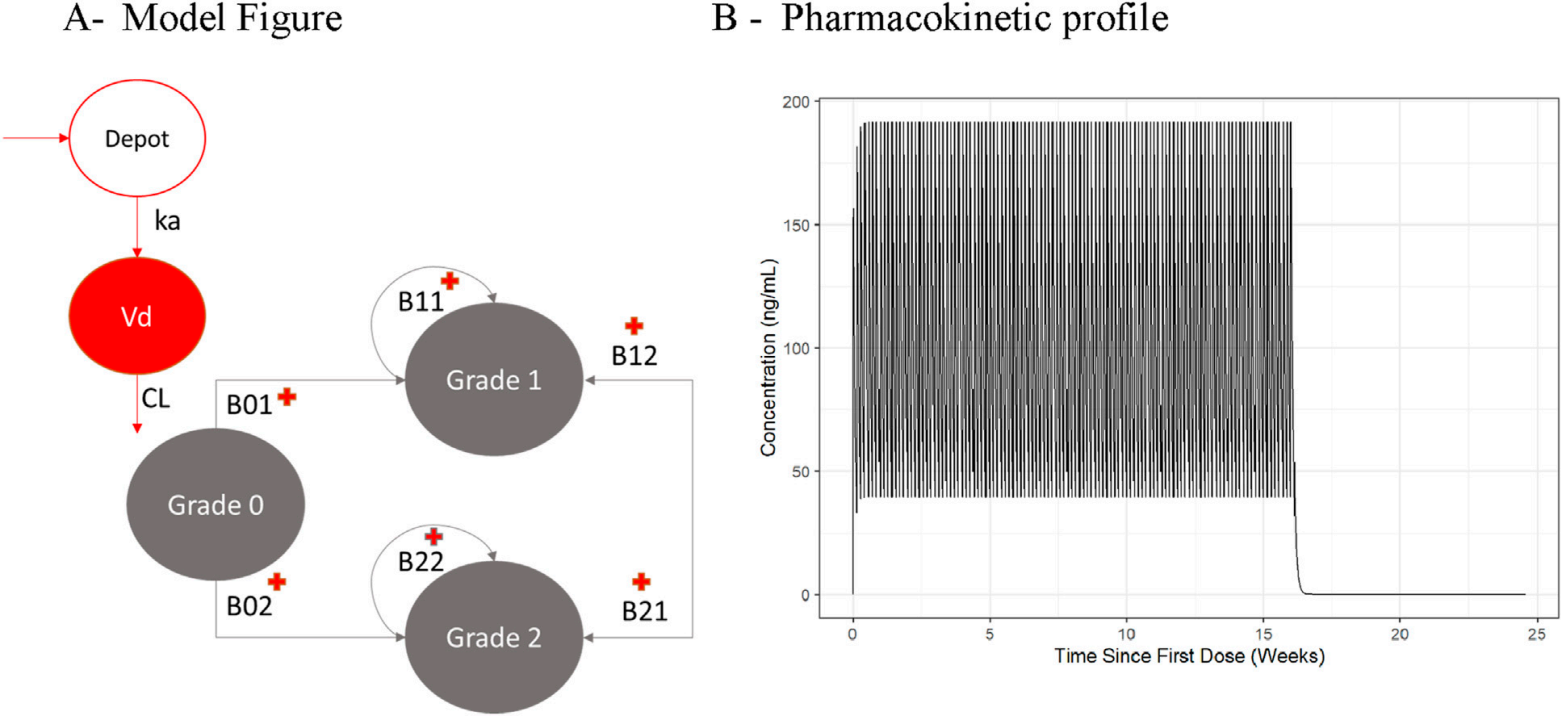
Illustration of the Population Pharmacokinetic and Proportional Odds Model used for the Motivation Example **(A)** and the resulting Pharmacokinetic profile **(B)**. Abbreviations: B01, B02, B11, B12, B21, B22 = transition parameters shown in Table 1. Reference source not found.; CL = clearance; Vd = central volume of distribution.

To illustrate the consequences of applying different derivations of C_avTE_ in subjects without events, we used a clinical trial dataset, de-identified and modified for ethical and confidentiality reasons. A simulation and estimation approach was used to ensure that only the derivations of C_avTE_ in censored subjects were changed. Exposures for virtual populations of different sizes (n = 50, 100 or 200) were simulated using a one compartment PopPK model with first-order elimination following administration of a 60 mg dose once daily for four dosing cycles of 28 days (Figure 1B). Events were simulated based on a proportional odds model with Markov components (Table 1). Grade 1 and 2 AEs were grouped into an “Any Grade” category, and only the first event for each virtual subject was selected. Grade 0 events were classified as “no events” and represented censored subjects. A schematic of the structural model is illustrated in Figure 1A. The logits of event probabilities were positively dependent on concentration in the central compartment. For subjects with an event, C_avTE_ was derived as the cumulative AUC divided by the time of the first event. For censored subjects (Grade 0), five imputation scenarios of the event time were explored to obtain the C_avTE_. These included EoT and EoT+7 days, +14 days, +21 days, and +28 days of follow up time. The magnitude of the ER relationship was modified by changing both Emax parameters from 0.25-fold to 1.25-fold in the same way. The Emax value assigned depended on the previous event score (either no event, or an event), with maximal drug effect implemented on a logit scale, as described previously (Zingmark et al., 2005).

**TABLE 1:** Parameter values used for the Example Scenario.

Description	Parameter	Estimate	IIV (CV%)
Parameters for the PopPK and proportional odds models			
Clearance (L/h)	CL	17.7	54.0
Volume (L)	V	229	34.1
Absorption rate constant (/h)	Ka	4.23	95.5
Lag time (h)	Alag	0.154	—
Transition from Grade 0 to Grade 1	B01	−6.59	10.0%
Transition from Grade 0 to Grade 2	B02	−1.80	10.0%
Transition from Grade 1 to Grade 1	B11	0.311	10.0%
Transition from Grade 1 to Grade 2	B12	−6.70	10.0%
Transition from Grade 2 to Grade 2	B22	−0.684	10.0%
Transition from Grade 2 to Grade 1	B21	−0.563	10.0%
Maximum effect when previous event grade = 0 (no event)	Emax_0_	4.73	10.0%
Maximum effect when previous event grade ≥ 1 (yes event)	Emax_1_	1.09	10.0%
Half maximal effective concentration	EC50 (ng/mL)	6.05	10.0%

Abbreviations: CV, coefficient of variation; IIV, interindividual variability; PopPK, population pharmacokinetic.

Once the data was simulated, a logistic regression analysis was used for the estimation using each of the five different C_avTE_ scenarios. C_avTE_ based ER models were compared to C_av,ss_ based ER models.

The logistic regression model was based on the following Equation 1:

$$logit\left(P_{i,responder}\right)=\log\left(\frac{P_{i,responder}}{1-P_{i,responder}}\right)=\beta_{0}+{\beta X}_{i},$$ (1)

where *P*_*i,responder*_ is the probability of the event of interest, *β*0 and *β* are scalar and vector parameters that represent the baseline logit and the effect of *X*_*i*_ (e.g., C_avTE_) on the logit, respectively.

### 2.2 Computation

All simulations were conducted using the mrgsolve package and logistic regression analyses were performed in R (version 4.3.0 within RStudio v. 2023.03.1).

## 3 Results

**FIGURE 2:**
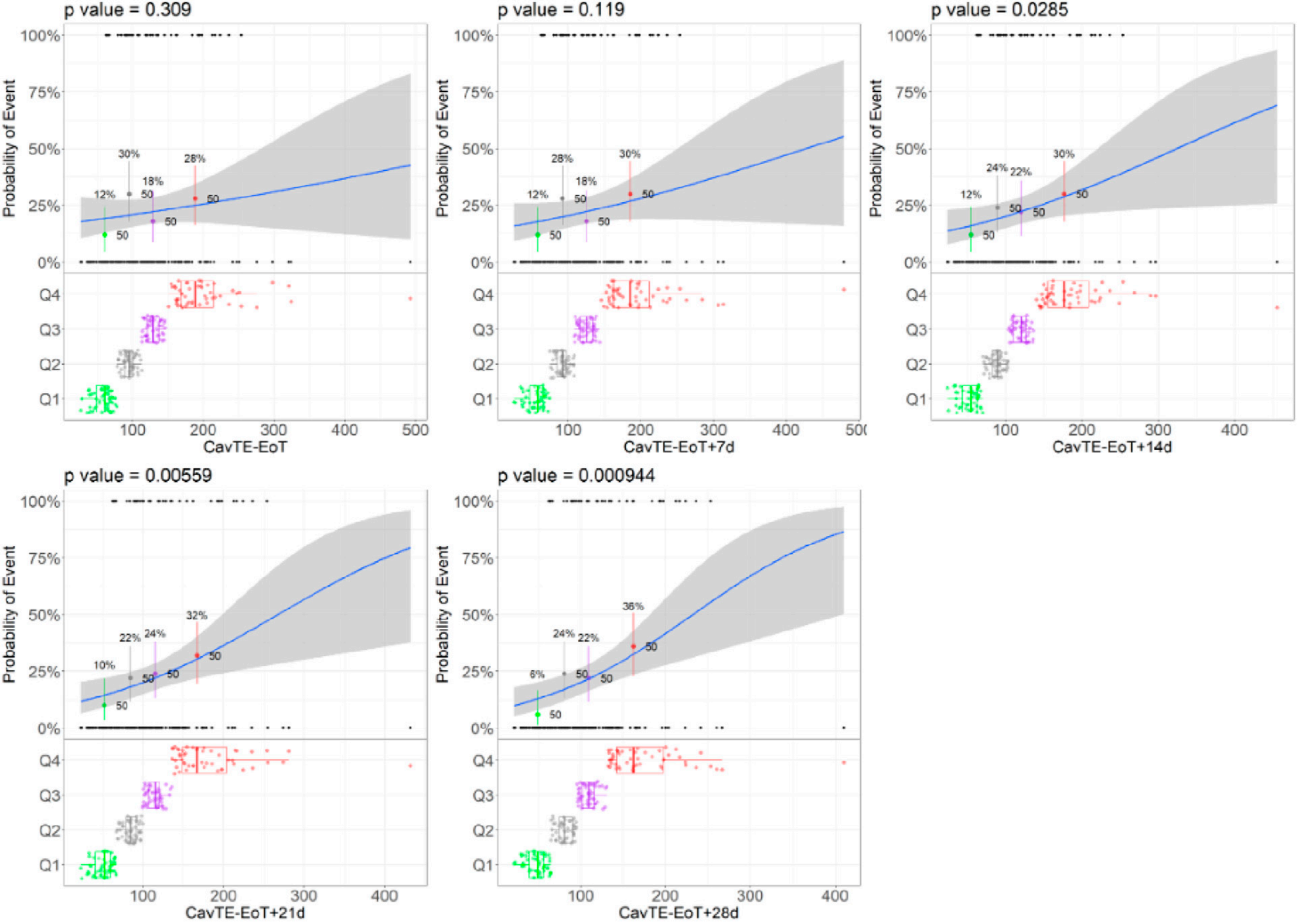
Trends of changes in the Logistic Regression Models illustrated for the Example Scenario. Abbreviations: EoT = end of treatment, C_avg,TE_ = average concentration to event or time-average exposure. Notes: Blue lines represent the logistic regression; grey shaded area represents 95% confidence interval; black dots represent the exposures with a corresponding event (0 no event, 1 yes event). The colored dots and boxplots represent exposure distributions for each quartile with boxplots presenting the median and the 95% CI of each quartile for all exposure metrics. The p-value presents the p-value corresponding to the slope parameter. Example scenario: Sample size = 100, CL = 17.7 L/h, and Emax = 0.5-fold.

For illustrative purposes the results for this case study are presented for an example scenario Emax of 0.5-fold using a sample size of 200 subjects (Figure 2). For all other scenarios tested, the same trends were observed as represented by the results shown below. Consistent event rates were generated across the three virtual study size scenarios (Supplementary Table S1) using the PopPK and proportional odds model with Markov components. As expected, higher event rates were obtained with models that included a higher Emax value. Exposure distributions are illustrated in Supplementary Figure S2 showing that an increase in the follow-up time (included in the calculation of C_avTE_) were associated with a left shift (i.e., lower exposure) in the C_avTE_ distribution for the censored subjects, also noted in the regression plots.

Across all different strengths of the ER relationships, driven by an increase in Emax, a consistent trend was demonstrated, irrespective of the sample size. Logistic regression ER models analyzing the simulated data presented a lower p-value on the slope (*β*) of the drug effect, with increasing times used to derive C_avTE_ in censored patients (Table 2). In addition, statistical significance (e.g., p < 0.05) on the p-value of slope (*β*, which represents the coefficient for exposure) was achieved at longer imputed times and increasing sample size (Figure 2; Table 2). Statistical significance (Table 2) was only achieved in the dataset with 50 subjects when EoT+21 or EoT+28 days follow-up was used. The change in the model slope was noticeably different across the five scenarios, with steepness increasing when increasing follow-up times were used to derive C_avTE_ (Supplementary Figure S1). The same trend was found across all tested ER relationships and is seen also in exploratory boxplot distribution plots (Supplementary Figure S3). Table 2 further shows that statistical power decreases when the number of subjects in the two categories (yes vs. no events) become imbalanced. An increased strength of the ER relationship (using an Emax change of 1.5-fold) resulted in <5% of subjects without an event (Supplementary Table S1), causing estimation difficulties due to substantial imbalances. Further, despite the strong relationship used in the simulation, the power to detect it is limited even with 200 subjects in the dataset.

**TABLE 2:** Significance of logistic regression exposure-response relationships, presented as p-values, based on imputation of time for censored events for the Scenario using a CL of 17.7 L/h.

Drug effect	C_avTE_				
	EoT	EoT + 7 days	EoT + 14 days	EoT + 21 days	EoT+ 28 days
Sample Size = 50					
0.25	0.839	0.675	0.493	0.347	0.236
0.5	0.575	0.399	0.237	0.134	0.0741
0.75	0.405	0.225	0.100	0.0437	0.0195
1	0.660	0.880	0.853	0.624	0.445
Sample Size = 100					
0.25	0.799	0.603	0.396	0.244	0.142
0.5	0.306	0.156	0.059	0.0201	0.00653
0.75	0.0862	0.0284	0.00687	0.00172	0.000465
1	0.0388	0.0189	0.00829	0.0040	0.00216
Sample Size = 200					
0.25	0.270	0.143	0.0578	0.0221	0.00827
0.5	0.309	0.119	0.0285	0.00559	0.000944
0.75	0.0167	0.00244	0.000156	0.00000933	0.000000644
1	0.00351	0.000671	0.0000893	0.0000137	0.00000258
1.25	0.258	0.174	0.107	0.0668	0.0426

Abbreviations: EoT = end of treatment, C_avTE_, average concentration to event or time-average exposure, C_av,ss_ = average concentration at steady-state.

Note: For sample size N = 50 and N = 100 with an Emax ≥1.25 insufficient subjects (<5%) without an event were in the dataset, consequently logistic regression analysis was not performed. p-values are colored according to: >0.05 = white, 0.05–0.01 = light grey, 0.01–0.005 = light orange, <0.005 = light red.

## 4 Discussion and conclusion

In this study, we investigated the impact of using time-averaged exposure when analyzing binary endpoints in a time-invariant logistic regression framework. These analyses are frequently used to quantify ER efficacy and safety relationships, with the aim of supporting therapeutic dose optimization in drug development programs (Ruiz-Garcia et al., 2023). This investigation is essential for sponsors seeking regulatory approval, to quantitatively support decision-making around dose projection, selection, and justification for risk-to-benefit ratio. ER analyses are applicable at various stages in development, using nonclinical and clinical data across different patient populations. Investigation of the C_avTE_ - event relationship is useful because it uses the actual dosing history for each subject, and therefore accounts for any dose changes within the duration of treatment. Our case study demonstrates that using C_avTE_ could introduce a potential bias when analyzing binary endpoint data due to the method of time imputation for C_avTE_ derivation in censored subjects. In our motivation example, we used various EoT and follow-up times (as is commonly used for regulatory submissions) to illustrate that altered time imputations may produce differences in the tested ER relationships. Furthermore, we show that bias in ER relationships involving time-averaged exposure may be influenced by the strength of the “true” drug effect. Limitations of C_avTE_ derivation (not illustrated here) occur also when different dosing regimens or titration strategies are used throughout a clinical trial. For example, ER using C_avTE_ would be biased when events predominantly occur within the first hours of a dosing interval (e.g., infusion related adverse reactions), and the EoT period has time units of weeks. The ER relationship would likely be significant, with a steep slope for exposure and expected to be different to ER trends using other exposure metrics. In this scenario, exposures within the first dosing interval or cycle might be more informative than consideration of the entire treatment period. Overall, one needs to evaluate pharmacological and biological plausibility and different analysis factors (e.g., the time of the events with respect to observational period, the level of dose reduction/interruption) to carefully consider and select the most appropriate exposure metric for the ER analysis.

The case study limitations include the proportional model used for simulations, which does not directly simulate the event times. The case example was performed without dose modification, as these are often due to individual circumstances or clinical decisions and cannot be easily incorporated into simulations. Other aspects that impact exposure (e.g., linear/non-linear pharmacokinetic, irregular dosing schedules, flat vs. weight-based dosing, target-mediated pharmacokinetic) were not investigated here.

It is recognized that although time-invariant logistic regression is relatively fast and efficient (especially for larger studies), it overlooks recurring events and does not take into account the time course of ER. This has the potential drawback of ignoring important elements like onset of effect or duration of effect. A more comprehensive and better suited analysis would consider the longitudinal ER relationships throughout the study duration, given that multiple events may occur. For this purpose, logistic regression incorporating a proportional odds model with Markov components or repeated TTE is more suitable due to the capacity for including time-varying exposure to reflect changes in dosing regimen, rather than relying on summary-level exposure metrics. However, a logistic regression framework is a more commonly used approach when the primary aim is to determine “probability” and not “time-to-” response for a given exposure. This will provide an understanding of how the probability of efficacy compares to that for safety, to describe the risk-to-benefit profile. Once this is established, decisions to select the optimal dose regimen are feasible, after which it becomes useful to look at the response over time. While use of time-averaged exposure in an ER logistic framework allows for comparison with first dose or SS exposures, this must be interpreted with caution, as illustrated above for infusion-related AE. The results of a logistic regression using SS metrics with a TTE analysis using time-averaged exposure cannot be compared.

In conclusion, this case study highlights bias and cautions when deriving C_avTE_ in censored subjects analyzing binary response data against other (SS and first dose) exposure metrics. Although standard practices have been described (Ruiz-Garcia et al., 2023), regulatory guidance documents are generally broad and potentially lack detail with regards to addressing recent advances in specific therapeutic areas. Rules and recommendations around using C_avTE_ and its derivation are absent. We propose a need for more examples from the community to learn when C_avTE_ is appropriate, followed with a white paper or an updating of regulatory guidance on the relevance of different exposure metrics, when justifying the optimal dose range in Phase 1 to 3 studies. Overall, we believe that C_avTE_ can be a useful exposure metrics in an ER analysis, when considered along with physiological or biological plausibility, the drug’s pharmacokinetic, and mechanism of action. Utilization of additional sensitivity analyses to justify the utility of using C_avTE_ in logistic regression analyses when defining risk-to-benefit margins should be performed as appropriate.

## 5 Study highlights

### 5.1 What is the current knowledge on the topic?

Exposure-response (ER) analysis of binary endpoint data is an essential part of model-informed drug development. Commonly used exposure metrics, recommended by regulators, include the maximum concentration, minimum concentration, and the area under the concentration-time curve (AUC) at steady-state (SS). Time-averaged exposure to event (C_avTE_) is additionally investigated because this metric can account for dose interruptions and modifications and is therefore requested for comparison against relationships using SS exposures. However, the derivation of C_avTE_ requires imputation when analyzed in a logistic framework using binary response data, since the time of event in censored subjects is unknown.

### 5.2 What question did this study address?

This study investigates the impact of using various imputation methods when deriving C_avTE_ in censored subjects for ER analyses of binary endpoint data.

### 5.3 What does this study add to our knowledge?

Consideration of potential bias that is introduced when using C_avTE_ in ER analyses of binary endpoints, and how this may influence key dosing decisions.

### 5.4 How might this change drug development, and/or therapeutics?

When using ER analyses to inform decisions around dose selection, justification, and confirmation, the exposure metrics used require careful consideration. Analysis and decision makers should apply caution when evaluatinging ER relationships that are based on time-averaged exposure.

## Data Availability

The original contributions presented in the study are included in the article/Supplementary Material, further inquiries can be directed to the corresponding author.

## Abbreviation

AE, adverse events; AUC, area under the concentration-time curve; AUC_ss_, area under the concentration-time curve at steady-state; C_av,ss_, average concentration at steady-state, AUC_ss_/Dosing Interval; C_avTE_, time-averaged concentration to event; EoT, end of treatment; ER, exposure-response; PopPK, population pharmacokinetics; SS, steady-state; TTE, time-to-event.

## References

[B3] Food and Drug Administration (2003). Guidance for industry - exposure-response relationships — study design, data analysis, and regulatory applications. Microsoft Word - 5341fnl.doc (fda.gov).

[B4] Hu C. Yao Z. Chen Y. Randazzo B. Zhang L. Xu Z. (2018). A comprehensive evaluation of exposure–response relationships in clinical trials: application to support guselkumab dose selection for patients with psoriasis. J. Pharmacokinet. Pharmacodyn. 45, 523–535. 10.1007/s10928-018-9581-1 29549540

[B5] Khandelwal A. Grisic A.-M. French J. Venkatakrishnan K. (2022). Pharmacometrics golems: exposure-response models in oncology. Clin. Pharmacol. Ther. 112, 941–945. 10.1002/cpt.2564 35286713

[B6] Overgaard R. V. Ingwersen S. H. Tornøe C. W. (2015). Establishing good practices for exposure-response analysis of clinical endpoints in drug development. CPT Pharmacometrics Syst. Pharmacol. 4 (10), 565–575. 10.1002/psp4.12015 26535157 PMC4625861

[B7] Patel K. Lin Y. W. Largajolli A. Edwards A. Y. Cheung S. Y. A. Hennig S. (2022). Impact of exposure metric on binary endpoints in exposure-response analysis. ACOP13. 10.3389/fphar.2024.1487062 PMC11780246 39885927

[B8] Ruiz-Garcia A. Baverel P. Bottino D. Dolton M. Feng Y. González-García I. (2023). A comprehensive regulatory and industry review of modeling and simulation practices in oncology clinical drug development. J. Pharmacokinet. Pharmacodyn. 50 (3), 147–172. 10.1007/s10928-023-09850-2 36870005 PMC10169901

[B9] Wang B. Deng R. Hennig S. Badovinac Crnjevic T. Kaewphluk M. Kagedal M. (2022). Population pharmacokinetic and exploratory exposure-response analysis of the fixed-dose combination of pertuzumab and trastuzumab for subcutaneous injection in patients with HER2-positive early breast cancer in the FeDeriCa study. Cancer Chemother. Pharmacol. 89 (4), 499–512. 10.1007/s00280-021-04296-0 34106303 PMC8187458

[B10] Wiens M. R. French J. L. Rogers J. A. (2023). Confounded exposure metrics. CPT Pharmacometrics Syst. Pharmacol. 00, 187–191. 10.1002/psp4.13074 PMC10864924 37984457

[B11] Yee K. L. Kleijn H. J. Kerbusch T. Matthews R. P. Dorr M. B. Garey K. W. (2019). Population pharmacokinetics and pharmacodynamics of bezlotoxumab in adults with primary and recurrent *Clostridium difficile* infection. Antimicrob. Agents Chemother. 63 (2), 019711–e2018. 10.1128/AAC.01971-18 PMC6355577 30455246

[B12] Yin O. Iwata H. Lin C. C. Tamura K. Watanabe J. Wada R. (2021b). Exposure-response relationships in patients with HER2-positive metastatic breast cancer and other solid tumors treated with trastuzumab deruxtecan. Clin. Pharmacol. Ther. 110 (4), 986–996. 10.1002/cpt.2291 33999422 PMC8518417

[B13] Yin O. Zahir H. French J. Polhamus D. Wang X. van de Sande M. (2021a). Exposure–response analysis of efficacy and safety for pexidartinib in patients with tenosynovial giant cell tumor. CPT Pharmacometrics and Syst. Pharmacol. 10 (11), 1422–1432. 10.1002/psp4.12712 PMC8592513 34585528

[B14] Zingmark P. H. Kågedal M. Karlsson M. O. (2005). Modelling a spontaneously reported side effect by use of a Markov mixed-effects model. J. Pharmacokinet. Pharmacodyn. 32 (2), 261–281. 10.1007/s10928-005-0021-7 16283538

