# Supplementary material for: Impact of using time-averaged exposure metrics on binary endpoints in exposure-response analyses
Source: Front Pharmacol. 2025 Jan 16;15:1487062. doi: 10.3389/fphar.2024.1487062 (PMC11780246; doi:10.3389/fphar.2024.1487062)
Supplement: Supplementary file 1 [file DataSheet1.docx]

Supplementary material

| Table S1 Summary of observed incidence rate across all scenarios | | | |
| --- | --- | --- | --- |
| Drug Effect | Incidence Rate (%) (95% CI) | | |
|  | N=50 | N=100 | N=200 |
| 0.25 | 12.0 (4.53 – 24.3) | 9.0 (4.20 – 16.4) | 8.5 (5.03 – 13.3) |
| 0.50 | 20.0 (10.0 – 33.7) | 21.0 (13.5 – 30.3) | 21.0 (15.6 – 27.3) |
| 0.75 | 52.0 (37.4 – 66.3) | 61.0 (50.7 – 70.6) | 49.5 (42.4 – 56.6) |
| 1.00 | 96.0 (73.3 – 94.2) | 89.0 (81.2 – 94.4) | 84.5 (78.7 – 89.2) |
| 1.25 | 100 (92.9 – 100) | 98.0 (93.0 – 99.8) | 96.5 (92.9 – 98.6) |
| 1.5 | 100 (92.2 – 100) | 100 (96.4 – 100) | 99.5 (97.2 – 100) |
| Abbreviations: N= number of virtual subjects in the dataset | | | |

Figure S1 Changes in logistic regression relationships with increase in imputed time values for deriving time-averaged concentrations in censored subjects


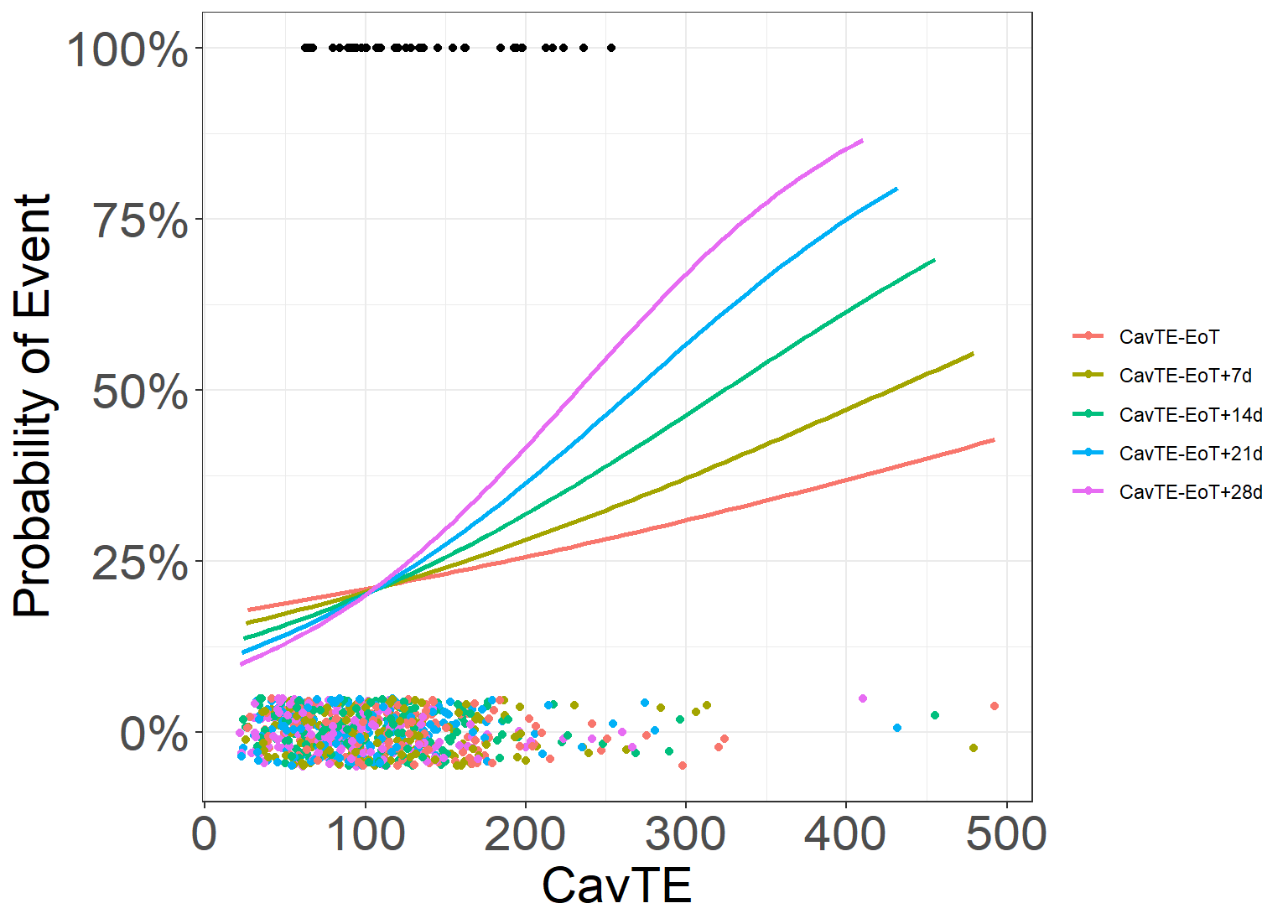


Abbreviations: CavTE = time-average concentration to event; d = day; EoT = end of treatment

Note: Lines illustrates the model fit of a logistic regression, black dots represent the exposures for subjects with an event, colored dots represent the exposures for censored subjects with changing imputed time used to derive CavTE.

Figure S2 Correlation and distribution of time-averaged concentrations for subjects with events and censored subjects


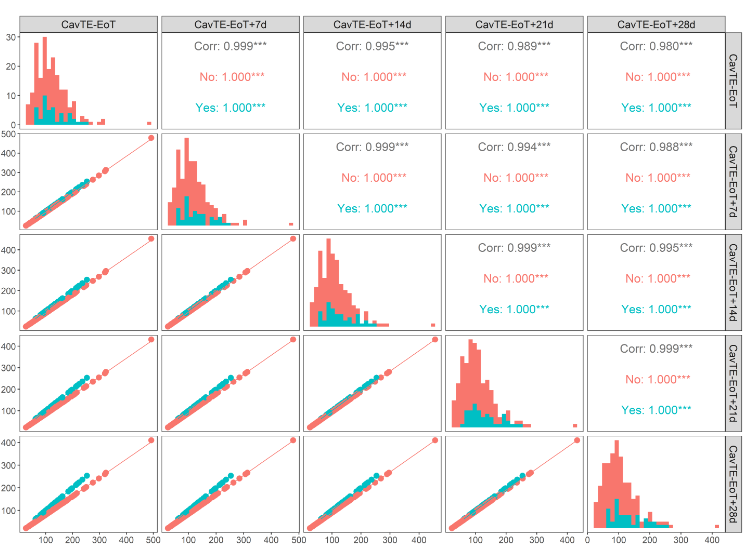


Abbreviations: C_avTE_ = time-average concentration to event; d = day; EoT = end of treatment

Note: red illustrates the subjects with an event and turquoise illustrates censored subjects.

Figure S3 Illustration of boxplot exposure distribution by yes/no event category (A) for the time of the first event vs time to steady-state (B) across the range of Emax values used.

| A | 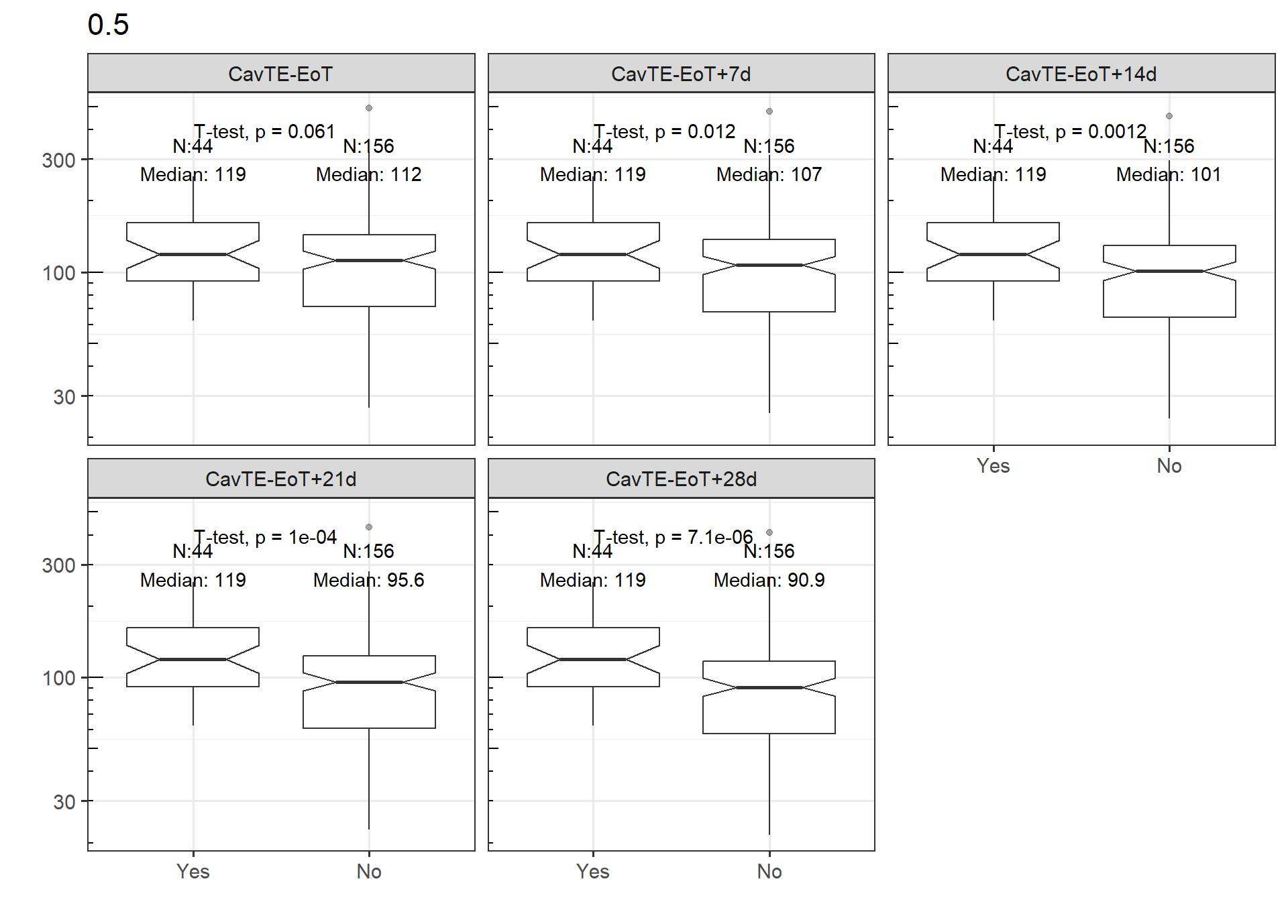 |
| --- | --- |
| B | 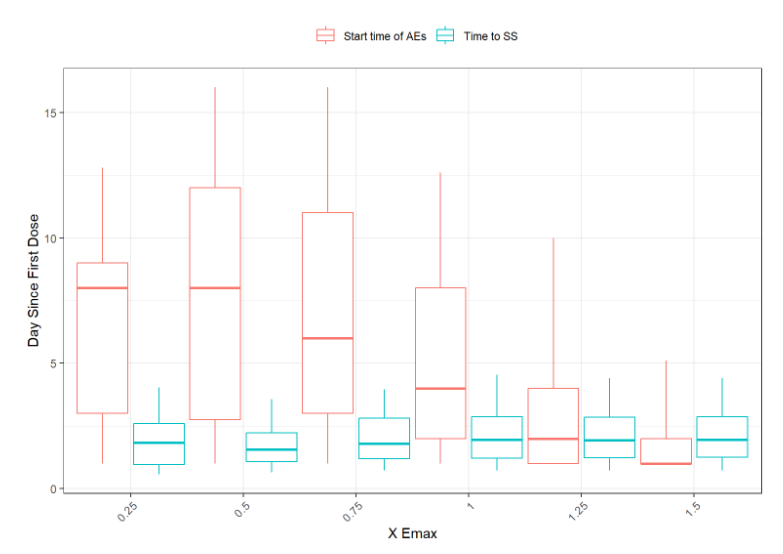 |

Abbreviations: AEs = Adverse events, CavTE = time-average concentration to event; d = day; EoT = end of treatment, SS = steady-state

Note: red illustrates time range to the start of an event and turquoise illustrates the time to steady-state.
